# Pneumoperitoneum without Intestinal Perforation in a Neonate: Case Report and Literature Review

**DOI:** 10.1155/2017/6907329

**Published:** 2017-04-16

**Authors:** Prabhavathi Gummalla, Gratias Mundakel, Maksim Agaronov, Haesoon Lee

**Affiliations:** ^1^Department of Pediatrics, SUNY Downstate Medical Center and Kings County Hospital Center, 450 Clarkson Avenue, Brooklyn, NY 11203, USA; ^2^Department of Pathology, Kings County Hospital Center, Brooklyn, NY, USA

## Abstract

Pneumoperitoneum in a preterm neonate usually indicates perforation of the intestine and is considered a surgical emergency. However, there are cases of pneumoperitoneum with no evidence of rupture of the intestine reported in the literature. We report a case of pneumoperitoneum with no intestinal perforation in a preterm neonate with respiratory distress syndrome who was on high frequency oscillatory ventilation (HFOV). He developed bilateral pulmonary interstitial emphysema with localized cystic lesion, likely localized pulmonary interstitial emphysema, and recurrent pneumothoraces. He was treated with dexamethasone to wean from the ventilator. Pneumoperitoneum developed in association with left sided pneumothorax following mechanical ventilation and cardiopulmonary resuscitation. Pneumoperitoneum resolved after the pneumothorax was resolved with chest tube drainage. He died from acute cardiorespiratory failure. At autopsy, there was no evidence of intestinal perforation. This case highlights the fact that pneumoperitoneum can develop secondary to pneumothorax and does not always indicate intestinal perforation or require exploratory laparotomy.

## 1. Case Report

A 560 gm male baby was born at 23^+6^ weeks of gestational age by spontaneous vaginal delivery to a 41-year-old mother with prolonged rupture of membranes for 48 hrs. Mother was treated with clindamycin and received two doses of betamethasone prior to delivery. The baby was hypotonic at birth and was noted to have poor respiratory effort. He was intubated and received the first dose of surfactant and was placed on synchronized intermittent mandatory ventilation (SIMV) with FiO_2_ 0.4 and transported to the neonatal intensive care unit. Initial chest roentgenogram (CXR) showed diffuse ground glass opacity suggestive of respiratory distress syndrome. He received the second dose of surfactant on first day of life. He was hypotensive and dopamine drip was started. He was treated with intravenous ampicillin and gentamicin for 48 hrs. Initial blood cultures were negative. He received a course of ibuprofen to facilitate PDA closure.

**Figure 1:**
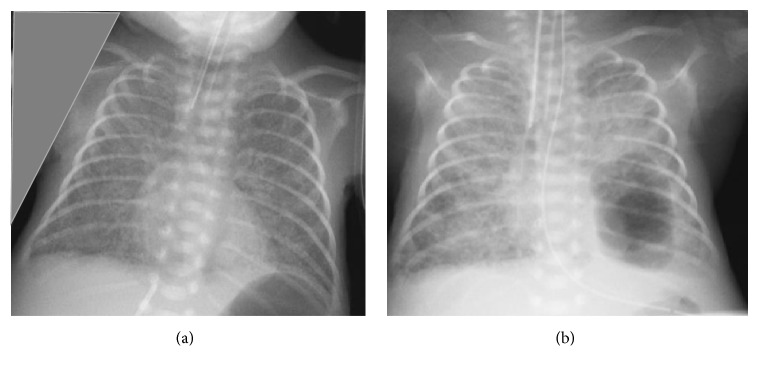
(a) CXR shows linear lucencies suggestive of PIE in both lungs on 6th day of life. (b) CXR shows bilateral PIE with cystic lesion in left lower lobe on 17th day of life.

On the third day of life, he developed pneumothorax on right side of the chest, which resolved spontaneously in 48 hrs. On the fifth day of life, he had recurrent oxygen desaturations and repeat CXR showed pulmonary interstitial emphysema (PIE) in both lungs (Figure 1(a)). On eleventh day of life, his respiratory status worsened requiring increased inspired oxygen concentration. CXR showed right side pneumothorax and cystic lesion in the left lower lobe, 8 mm in diameter. A chest tube was inserted on the right side and the pneumothorax resolved in 72 hrs. The cystic lesion in the left lower lobe gradually increased in size to 33 mm in diameter in the next two weeks (Figure 1(b)). His oxygen requirements increased gradually to FiO_2_ 0.9–1.0 on SIMV with high inspiratory pressures.

**Figure 2:**
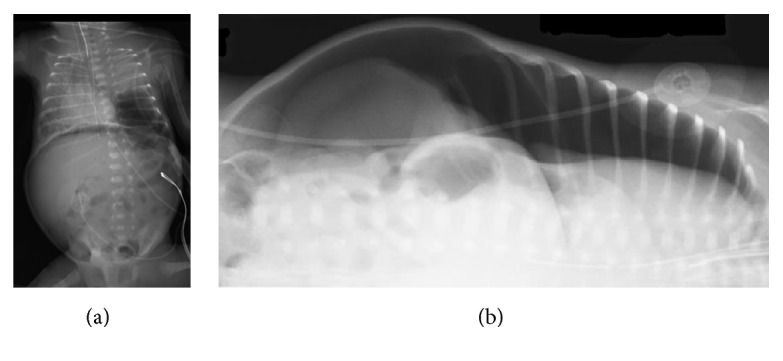
(a) Radiograph of chest and abdomen shows pneumothorax, pneumoperitoneum, and cystic lesion in the left lower lobe. (b) Right lateral decubitus view of chest and abdomen shows left sided pneumothorax with pneumoperitoneum.

On nineteenth day of life, he was switched to high frequency oscillatory ventilation with inhaled nitric oxide 20 ppm to improve oxygenation and prevent further air leak. He was started on intravenous dexamethasone. Three hours after administration of dexamethasone, he suddenly became apneic and bradycardic with oxygen desaturation to 20% on HFOV with FiO_2_ 0.8, mean airway pressure (MAP) 11 cm H_2_O, delta P 24, and frequency 14.5 Hz with inhaled nitric oxide 19 ppm. He had cardiopulmonary resuscitation (CPR) with chest compression. His blood gases showed significant respiratory acidosis. He developed tension pneumothorax on left side with pulmonary interstitial emphysema and pneumoperitoneum (Figures 2(a) and 2(b)). His abdomen was distended but remained soft with bowel sounds. He had emergent chest tube placement. A Penrose drain was inserted into abdomen which drained minimal peritoneal fluid.

**Figure 3:**
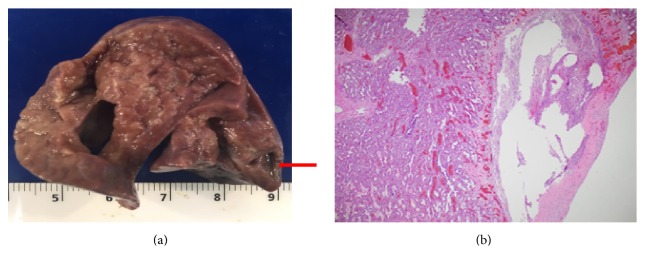
(a) Gross image of the left lung with 4 mm cystic lesion in left lower lobe. (b) Histology of left lower lobe cystic lesion lined by inflammatory cells and fibrosis.

During the next few days, he improved and HFOV was switched to synchronized intermittent mandatory ventilation (SIMV) with FiO_2_ 0.4. He was started on enteral feeds and he tolerated them well. Next day, he had acute deterioration with oxygen desaturations, bradycardia, and hypotension. He had significant respiratory and metabolic acidosis on blood gases. CXR showed right upper lobe atelectasis. His SIMV was switched back to HFOV. He was noted to be anemic and was transfused with 15 mls/kg of packed red blood cells. He remained hypotensive with poor perfusion on dopamine and epinephrine infusions. He was treated with intravenous meropenem and linezolid to treat presumed sepsis. He had distended abdomen and abdominal X-ray showed dilated loop of transverse colon with no evidence of intra-abdominal free air. Blood cultures were negative at 5 days. He died from cardiorespiratory failure. Autopsy confirmed premature lungs appropriate for gestational age, pulmonary interstitial emphysema in both lungs, 4 mm cystic lesion lined by fibrous tissue in left lower lobe which may represent localized pulmonary interstitial emphysema (Figures 3(a) and 3(b)), and congested and dilated loop of transverse colon with no macroscopic or microscopic evidence of perforation or pneumatosis intestinalis. Diaphragm was intact. Both bacterial and viral cultures of tissues and fluids at autopsy were negative.

## 2. Discussion 

Pneumoperitoneum develops from perforation of hollow viscus in 85–95% of cases [1–4]. Most common cause of pneumoperitoneum in a preterm baby is necrotizing enterocolitis. Other causes of pneumoperitoneum include isolated gastric, duodenal, colonic, and rectal perforations. Pneumoperitoneum can also be a complication of cardiopulmonary resuscitation, mechanical ventilation, peritoneal dialysis, endoscopic procedures, previous abdominal surgeries, and pneumatosis cystoides intestinalis due to clostridium perfringens [1, 5]. 97% cases of postoperative free air resolve within 5 days [6]. Khan et al. [7] reported on 89 neonates with pneumoperitoneum, 51% of which were due to necrotizing enterocolitis, 49% unrelated to necrotizing enterocolitis and 7% with no apparent cause with benign pneumoperitoneum. In the pediatric population, pneumoperitoneum occurs in 1–3% of infants who are mechanically ventilated [5].

This case describes a pneumoperitoneum and pneumothorax that occurred in a neonate who had respiratory distress syndrome and ventilated with high frequency oscillation after cardiopulmonary resuscitation. We postulate that bilateral pulmonary interstitial emphysema with localized cystic lesion in the left lower lobe in addition to neonatal respiratory distress syndrome in this case led to stiff lung with decreased lung compliance. We also postulate that the cystic lesion in the left lower lobe may represent localized form of pulmonary interstitial emphysema. Mechanical ventilation probably caused alveolar wall stretching and rupture producing tension pneumothorax. Rupture of localized cystic lesion in the left lower lobe may have contributed to the tension pneumothorax and air under pressure may have travelled along the normal anatomical openings in the diaphragm, that is, aortic, inferior vena caval, and esophageal openings into the peritoneal cavity producing pneumoperitoneum. No evidence of intestinal perforation was found at autopsy. There was no subcutaneous emphysema, pneumomediastinum, or retroperitoneal free air that could have gained entrance to the peritoneal cavity in this case.

The physiological mechanism of air leak of ruptured lung was first demonstrated by Macklin [8] in 1939, in which they described that air from ruptured alveoli escaped into the pulmonary interstitial tissues and travels along the sheaths of pulmonary vessels to the root of the lung and there into the mediastinum and further into the pleural cavity resulting in pneumomediastinum and pneumothorax. In the case of pneumoperitoneum, air can pass through the normal anatomical openings in the diaphragm, that is, aortic, esophageal, inferior vena caval openings, splanchnic nerves, roots of azygos system of veins, and the sympathetic chain. It can also pass through a posterolateral defect of the diaphragm secondary to arrest in the closure of pleuroperitoneal canal and diaphragmatic defects at the sternocostal and lumbocostal region as described by Leininger et al. [9] in a case of tension pneumoperitoneum and pneumothorax in the newborn. Aranda [10] reported a neonate with hyaline membrane disease, bilateral intrapulmonary interstitial emphysema, bilateral tension pneumothorax, and massive pneumomediastinum who later developed pneumoperitoneum. Leonidas et al. [11] identified 9 cases of pneumoperitoneum in 222 mechanically ventilated new born infants, 4 of whom had no apparent bowel perforation and two had negative laparotomy for rupture of intestine.

Development of pneumoperitoneum in pneumothorax was described by few authors [12–15]. Campbell et al. [16] described three cases of early neonatal pneumoperitoneum from massive pneumomediastinum. Karagol et al. [17] reported a case of tension pneumothorax associated pneumoretroperitoneum and perirenal air in an extremely low birth weight infant. Köklü et al. [18] described a 34-week preterm baby, who was on mechanical ventilation, was extubated and discharged, and later developed pneumoperitoneum.

Air leak syndrome can occur in 1-2% of newborn and in up to 40% in meconium aspiration syndrome [19]. Risk factors for air leak syndrome are prematurity, very low birth weight, positive pressure ventilation with ventilatory parameters of high positive end expiratory pressure (PEEP), long inspiratory time, large tidal volumes, respiratory distress syndrome, meconium aspiration syndrome, congenital pneumonia, and pulmonary hypoplasia. Uneven ventilation in the lung results in atelectasis in some areas and air trapping in the other parts of the lung. Rapid changes in airway pressure during phasic change in ventilation can cause shear mechanism in the nonuniform lung, between the stiff segment and adjacent compliant lung resulting in alveolar rupture and air leak. Active Hering-Breuer expiratory reflex in a baby on the ventilator during inhalation phase can result in air leaks [20]. Displacement of endotracheal tube (ETT) into the right main bronchus is associated with increased risk of pneumothorax. Perforation of the lung from suction catheters that passed through endotracheal tube rarely resulted in pneumothorax. Air leaks in neonates have significantly decreased due to the current use of surfactant for immature lungs, strategic use of permissive hypercapnia, high frequency low tidal volume ventilator modality, and use of neuromuscular blocking agents.

Klinger et al. [21] reported the pneumothorax in very low birth weight infants associated with the factors present on the day that pneumothorax occurred and the initial severity of lung disease. They suggested that minimizing peak inspiratory pressures and use of optimal PEEP to decrease the risk of pneumothorax in very low birth weight (VLBW) infants. Stevens et al. [22] demonstrated that the use of surfactant early with rapid extubation to nasal continuous positive airway pressure (NCPAP) may reduce the risk of air leak syndrome. Morley et al. [23] reported 9% incidence of pneumothorax after use of CPAP in preterm babies. High frequency oscillatory ventilation was thought to reduce the incidence of air leak syndrome [20]. A meta-analysis by Cools et al. [24] involving 10 trials with 3229 participants did not find decreased incidence of pulmonary air leaks in premature babies with respiratory distress syndrome, who were ventilated with high frequency oscillatory ventilation. Keszler et al. [25] reported that high frequency jet ventilation helped more rapid resolution of pulmonary interstitial emphysema than conventional mechanical ventilation (CMV) but had no significant difference in the outcome analysis of new air leaks.

A thorough clinical examination aided by laboratory evidence and radiological imaging (football sign in case of NEC) of contrast studies may help to differentiate surgical and nonsurgical cause of pneumoperitoneum. Some studies attempted to distinguish pneumoperitoneum secondary to gastrointestinal perforations from that caused by pulmonary air leak [12, 26–29] which suggest that abdominal X-ray films with patient in upright position and opacification of gastrointestinal tract with water soluble contrast material should be carried out in every newborn with pneumoperitoneum and pulmonary disease especially on ventilator. Vanhaesebrouck et al. described measurement of P0_2_ in the peritoneal air by abdominal paracentesis that was helpful in differentiating between the two [30]. In those with pneumoperitoneum secondary to pneumothorax, partial pressure of oxygen delivered by the ventilator to the lung was well above the partial pressure of the gas in the peritoneum leaked out of perforated bowel, which is essentially swallowed room air.

## 3. Conclusion

Pneumoperitoneum in extremely low birth weight neonates is most likely due to intestinal perforation and it needs to be ruled out clinically and radiographically. In those babies who are mechanically ventilated and have developed air leak syndromes, it is important to remember the possibility of the caudal progression of pneumothorax air through the diaphragmatic openings to cause pneumoperitoneum, which resolves once pneumothorax is managed appropriately with chest tube placement, thus avoiding exploratory laparotomy. Gentle ventilation with high frequency, low tidal volumes, low pressure strategy, and permissive hypercapnia are the important practices to reduce incidence of air leak syndrome in neonates requiring mechanical ventilation.
